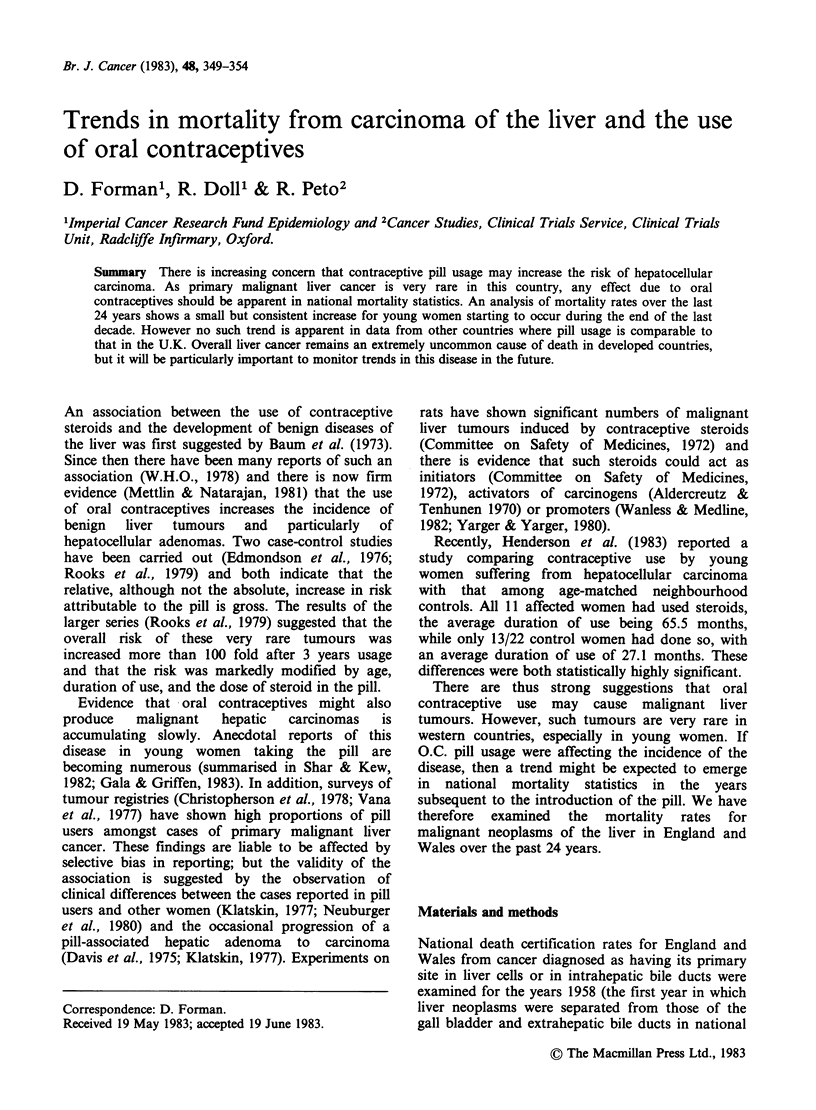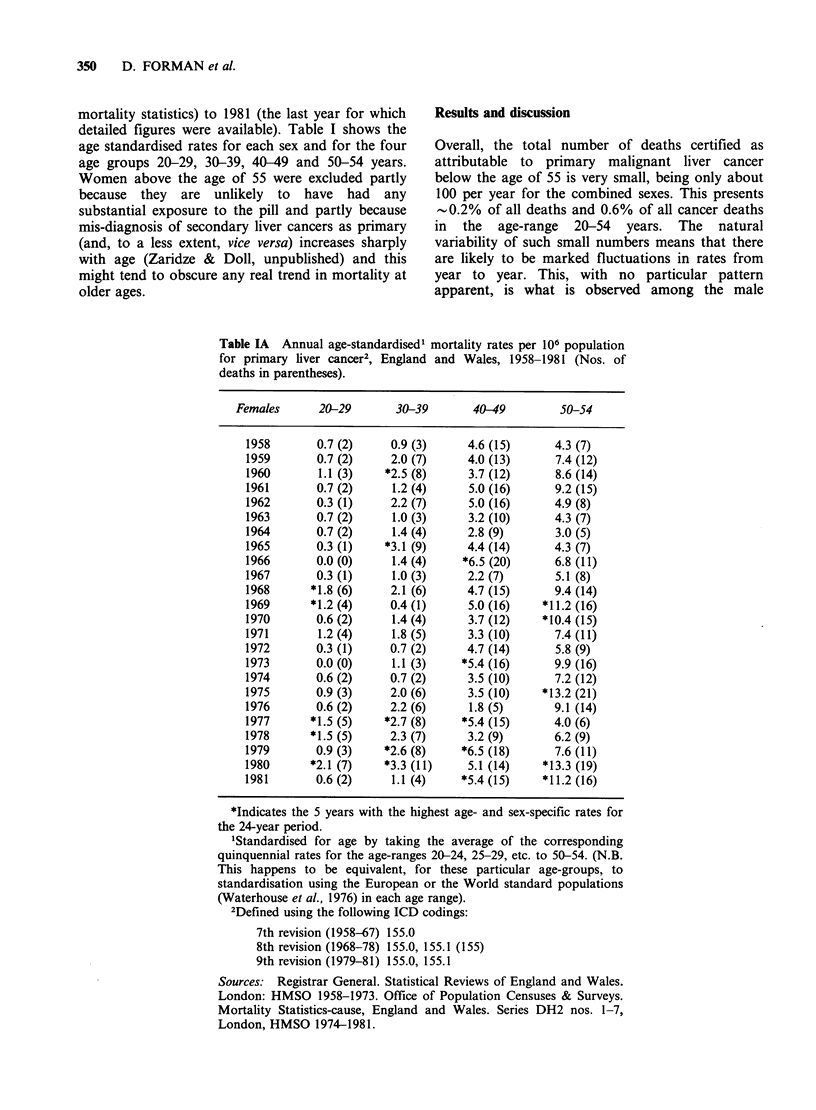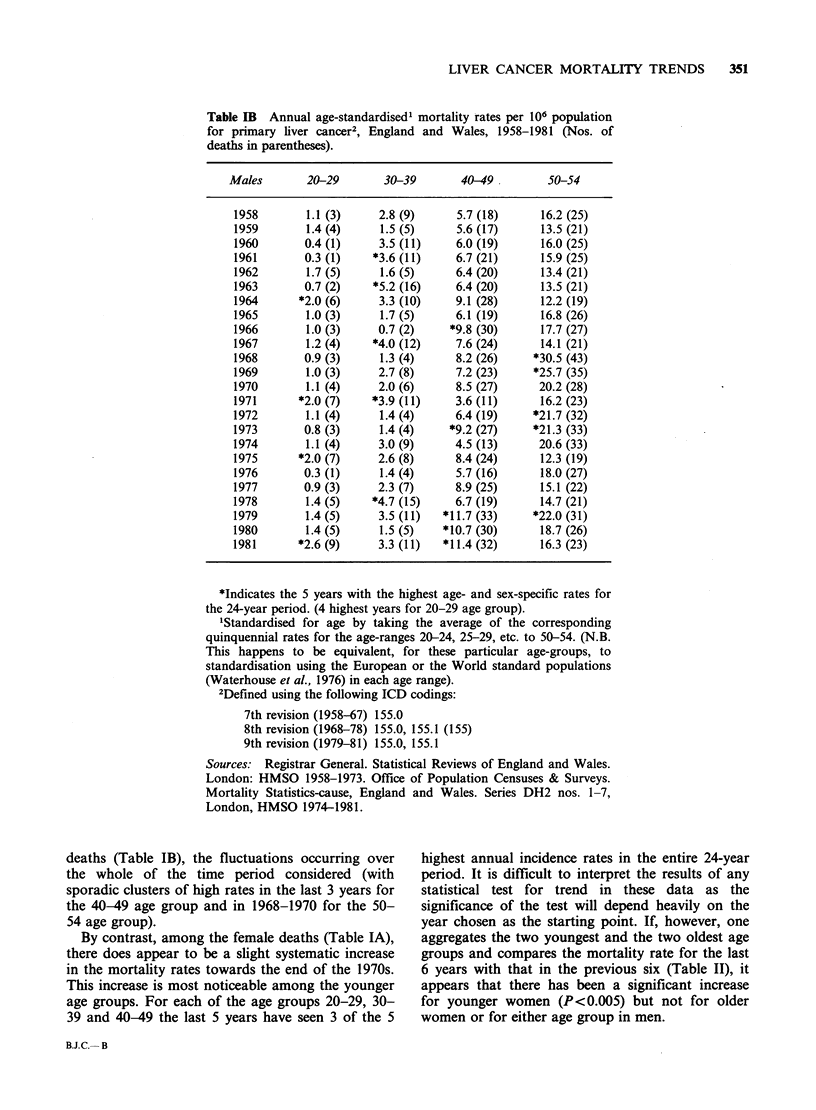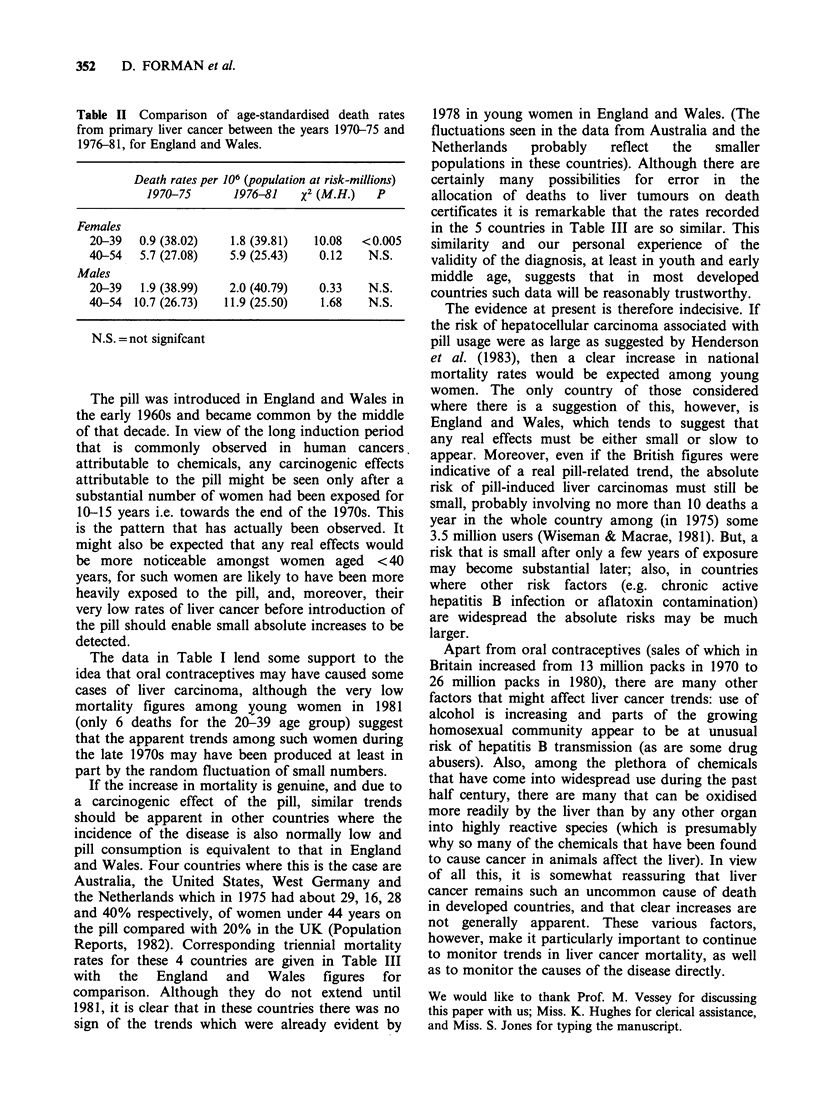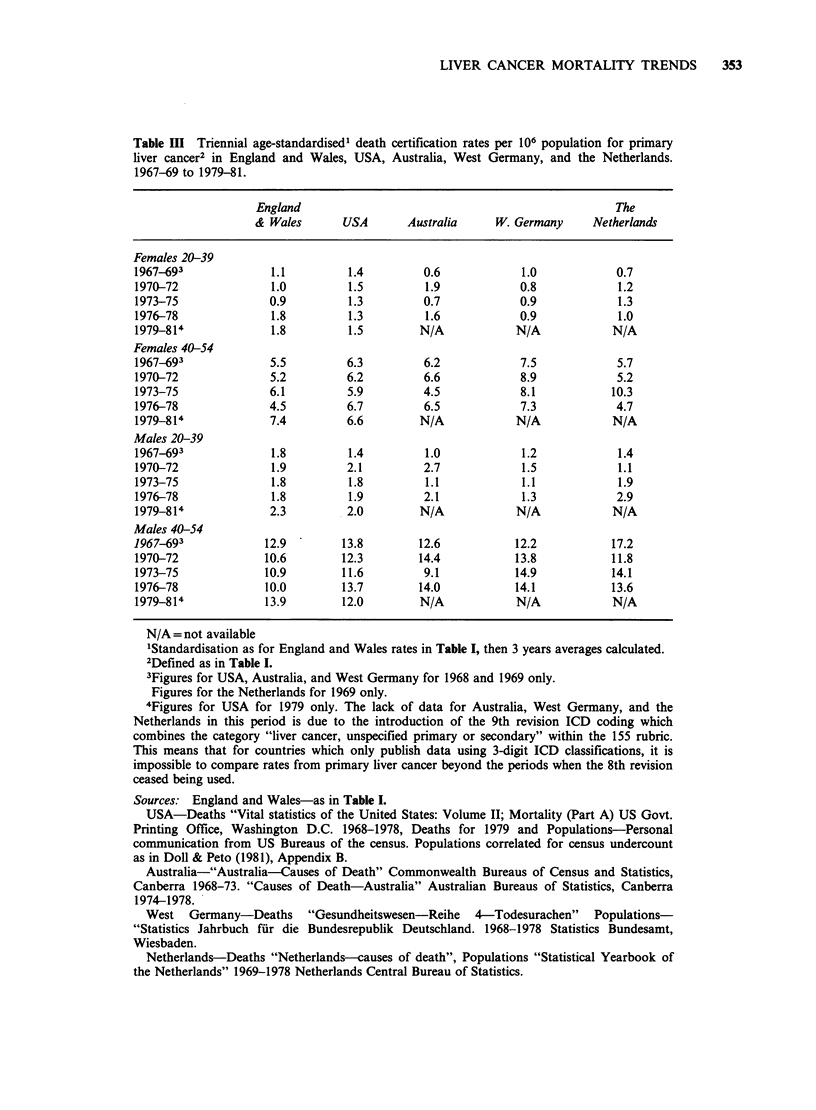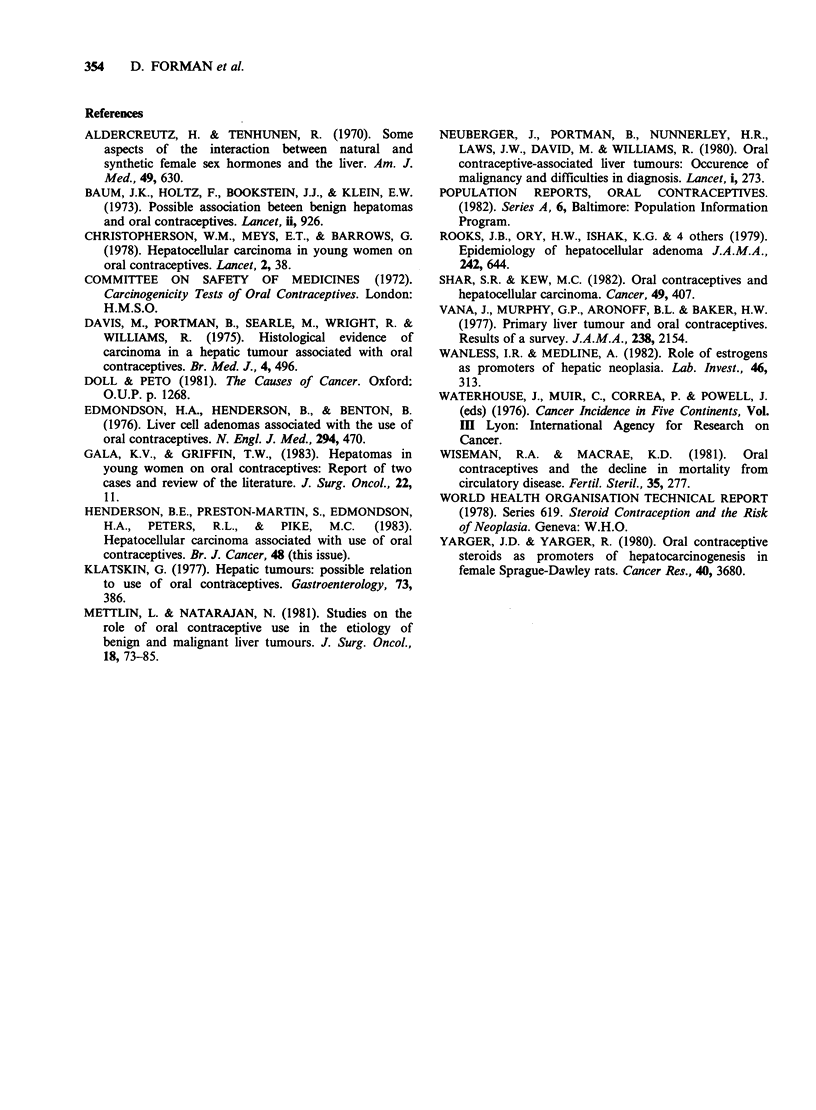# Trends in mortality from carcinoma of the liver and the use of oral contraceptives.

**DOI:** 10.1038/bjc.1983.199

**Published:** 1983-09

**Authors:** D. Forman, R. Doll, R. Peto

## Abstract

There is increasing concern that contraceptive pill usage may increase the risk of hepatocellular carcinoma. As primary malignant liver cancer is very rare in this country, any effect due to oral contraceptives should be apparent in national mortality statistics. An analysis of mortality rates over the last 24 years shows a small but consistent increase for young women starting to occur during the end of the last decade. However no such trend is apparent in data from other countries where pill usage is comparable to that in the U.K. Overall liver cancer remains an extremely uncommon cause of death in developed countries, but it will be particularly important to monitor trends in this disease in the future.


*Br. J. Cancer* (1983), **48**, 349–354

### Trends in mortality from carcinoma of the liver and the use of oral contraceptives

D. Forman[1], R. Doll[1] & R. Peto[2]

[1]*Imperial Cancer Research Fund Epidemiology and* [2]*Cancer Studies, Clinical Trials Service, Clinical Trials Unit, Radcliffe Infirmary, Oxford.*

**Summary** There is increasing concern that contraceptive pill usage may increase the risk of hepatocellular carcinoma. As primary malignant liver cancer is very rare in this country, any effect due to oral contraceptives should be apparent in national mortality statistics. An analysis of mortality rates over the last 24 years shows a small but consistent increase for young women starting to occur during the end of the last decade. However no such trend is apparent in data from other countries where pill usage is comparable to that in the U.K. Overall liver cancer remains an extremely uncommon cause of death in developed countries, but it will be particularly important to monitor trends in this disease in the future.

An association between the use of contraceptive steroids and the development of benign diseases of the liver was first suggested by Baum *et al.* (1973). Since then there have been many reports of such an association (W.H.O., 1978) and there is now firm evidence (Mettlin & Natarajan, 1981) that the use of oral contraceptives increases the incidence of benign liver tumours and particularly of hepatocellular adenomas. Two case-control studies have been carried out (Edmondson *et al.*, 1976; Rooks *et al.*, 1979) and both indicate that the relative, although not the absolute, increase in risk attributable to the pill is gross. The results of the larger series (Rooks *et al.*, 1979) suggested that the overall risk of these very rare tumours was increased more than 100 fold after 3 years usage and that the risk was markedly modified by age, duration of use, and the dose of steroid in the pill.

Evidence that oral contraceptives might also produce malignant hepatic carcinomas is accumulating slowly. Anecdotal reports of this disease in young women taking the pill are becoming numerous (summarised in Shar & Kew, 1982; Gala & Griffen, 1983). In addition, surveys of tumour registries (Christopherson *et al.*, 1978; Vana *et al.*, 1977) have shown high proportions of pill users amongst cases of primary malignant liver cancer. These findings are liable to be affected by selective bias in reporting; but the validity of the association is suggested by the observation of clinical differences between the cases reported in pill users and other women (Klatskin, 1977; Neuburger *et al.*, 1980) and the occasional progression of a pill-associated hepatic adenoma to carcinoma (Davis *et al.*, 1975; Klatskin, 1977). Experiments on rats have shown significant numbers of malignant liver tumours induced by contraceptive steroids (Committee on Safety of Medicines, 1972) and there is evidence that such steroids could act as initiators (Committee on Safety of Medicines, 1972), activators of carcinogens (Aldercreutz & Tenhunen 1970) or promoters (Wanless & Medline, 1982; Yarger & Yarger, 1980).

Recently, Henderson *et al.* (1983) reported a study comparing contraceptive use by young women suffering from hepatocellular carcinoma with that among age-matched neighbourhood controls. All 11 affected women had used steroids, the average duration of use being 65.5 months, while only 13/22 control women had done so, with an average duration of use of 27.1 months. These differences were both statistically highly significant.

There are thus strong suggestions that oral contraceptive use may cause malignant liver tumours. However, such tumours are very rare in western countries, especially in young women. If O.C. pill usage were affecting the incidence of the disease, then a trend might be expected to emerge in national mortality statistics in the years subsequent to the introduction of the pill. We have therefore examined the mortality rates for malignant neoplasms of the liver in England and Wales over the past 24 years.

### Materials and methods

National death certification rates for England and Wales from cancer diagnosed as having its primary site in liver cells or in intrahepatic bile ducts were examined for the years 1958 (the first year in which liver neoplasms were separated from those of the gall bladder and extrahepatic bile ducts in national

Correspondence: D. Forman.
Received 19 May 1983; accepted 19 June 1983.

© The Macmillan Press Ltd., 1983

mortality statistics) to 1981 (the last year for which detailed figures were available). Table I shows the age standardised rates for each sex and for the four age groups 20–29, 30–39, 40–49 and 50–54 years. Women above the age of 55 were excluded partly because they are unlikely to have had any substantial exposure to the pill and partly because mis-diagnosis of secondary liver cancers as primary (and, to a less extent, *vice versa*) increases sharply with age (Zaridze & Doll, unpublished) and this might tend to obscure any real trend in mortality at older ages.

### Results and discussion

Overall, the total number of deaths certified as attributable to primary malignant liver cancer below the age of 55 is very small, being only about 100 per year for the combined sexes. This presents ~0.2% of all deaths and 0.6% of all cancer deaths in the age-range 20–54 years. The natural variability of such small numbers means that there are likely to be marked fluctuations in rates from year to year. This, with no particular pattern apparent, is what is observed among the male

**Table IA** Annual age-standardised[1] mortality rates per $10^6$ population for primary liver cancer[2], England and Wales, 1958–1981 (Nos. of deaths in parentheses).

| *Females* | *20–29* | *30–39* | *40–49* | *50–54* |
|---|---|---|---|---|
| 1958 | 0.7 (2) | 0.9 (3) | 4.6 (15) | 4.3 (7) |
| 1959 | 0.7 (2) | 2.0 (7) | 4.0 (13) | 7.4 (12) |
| 1960 | 1.1 (3) | *2.5 (8) | 3.7 (12) | 8.6 (14) |
| 1961 | 0.7 (2) | 1.2 (4) | 5.0 (16) | 9.2 (15) |
| 1962 | 0.3 (1) | 2.2 (7) | 5.0 (16) | 4.9 (8) |
| 1963 | 0.7 (2) | 1.0 (3) | 3.2 (10) | 4.3 (7) |
| 1964 | 0.7 (2) | 1.4 (4) | 2.8 (9) | 3.0 (5) |
| 1965 | 0.3 (1) | *3.1 (9) | 4.4 (14) | 4.3 (7) |
| 1966 | 0.0 (0) | 1.4 (4) | *6.5 (20) | 6.8 (11) |
| 1967 | 0.3 (1) | 1.0 (3) | 2.2 (7) | 5.1 (8) |
| 1968 | *1.8 (6) | 2.1 (6) | 4.7 (15) | 9.4 (14) |
| 1969 | *1.2 (4) | 0.4 (1) | 5.0 (16) | *11.2 (16) |
| 1970 | 0.6 (2) | 1.4 (4) | 3.7 (12) | *10.4 (15) |
| 1971 | 1.2 (4) | 1.8 (5) | 3.3 (10) | 7.4 (11) |
| 1972 | 0.3 (1) | 0.7 (2) | 4.7 (14) | 5.8 (9) |
| 1973 | 0.0 (0) | 1.1 (3) | *5.4 (16) | 9.9 (16) |
| 1974 | 0.6 (2) | 0.7 (2) | 3.5 (10) | 7.2 (12) |
| 1975 | 0.9 (3) | 2.0 (6) | 3.5 (10) | *13.2 (21) |
| 1976 | 0.6 (2) | 2.2 (6) | 1.8 (5) | 9.1 (14) |
| 1977 | *1.5 (5) | *2.7 (8) | *5.4 (15) | 4.0 (6) |
| 1978 | *1.5 (5) | 2.3 (7) | 3.2 (9) | 6.2 (9) |
| 1979 | 0.9 (3) | *2.6 (8) | *6.5 (18) | 7.6 (11) |
| 1980 | *2.1 (7) | *3.3 (11) | 5.1 (14) | *13.3 (19) |
| 1981 | 0.6 (2) | 1.1 (4) | *5.4 (15) | *11.2 (16) |

*Indicates the 5 years with the highest age- and sex-specific rates for the 24-year period.

[1]Standardised for age by taking the average of the corresponding quinquennial rates for the age-ranges 20–24, 25–29, etc. to 50–54. (N.B. This happens to be equivalent, for these particular age-groups, to standardisation using the European or the World standard populations (Waterhouse *et al.*, 1976) in each age range).

[2]Defined using the following ICD codings:

7th revision (1958–67) 155.0
8th revision (1968–78) 155.0, 155.1 (155)
9th revision (1979–81) 155.0, 155.1

*Sources:* Registrar General. Statistical Reviews of England and Wales. London: HMSO 1958–1973. Office of Population Censuses & Surveys. Mortality Statistics-cause, England and Wales. Series DH2 nos. 1–7, London, HMSO 1974–1981.

**Table IB** Annual age-standardised[1] mortality rates per $10^6$ population for primary liver cancer[2], England and Wales, 1958–1981 (Nos. of deaths in parentheses).

| *Males* | *20–29* | *30–39* | *40–49* | *50–54* |
|---|---|---|---|---|
| 1958 | 1.1 (3) | 2.8 (9) | 5.7 (18) | 16.2 (25) |
| 1959 | 1.4 (4) | 1.5 (5) | 5.6 (17) | 13.5 (21) |
| 1960 | 0.4 (1) | 3.5 (11) | 6.0 (19) | 16.0 (25) |
| 1961 | 0.3 (1) | *3.6 (11) | 6.7 (21) | 15.9 (25) |
| 1962 | 1.7 (5) | 1.6 (5) | 6.4 (20) | 13.4 (21) |
| 1963 | 0.7 (2) | *5.2 (16) | 6.4 (20) | 13.5 (21) |
| 1964 | *2.0 (6) | 3.3 (10) | 9.1 (28) | 12.2 (19) |
| 1965 | 1.0 (3) | 1.7 (5) | 6.1 (19) | 16.8 (26) |
| 1966 | 1.0 (3) | 0.7 (2) | *9.8 (30) | 17.7 (27) |
| 1967 | 1.2 (4) | *4.0 (12) | 7.6 (24) | 14.1 (21) |
| 1968 | 0.9 (3) | 1.3 (4) | 8.2 (26) | *30.5 (43) |
| 1969 | 1.0 (3) | 2.7 (8) | 7.2 (23) | *25.7 (35) |
| 1970 | 1.1 (4) | 2.0 (6) | 8.5 (27) | 20.2 (28) |
| 1971 | *2.0 (7) | *3.9 (11) | 3.6 (11) | 16.2 (23) |
| 1972 | 1.1 (4) | 1.4 (4) | 6.4 (19) | *21.7 (32) |
| 1973 | 0.8 (3) | 1.4 (4) | *9.2 (27) | *21.3 (33) |
| 1974 | 1.1 (4) | 3.0 (9) | 4.5 (13) | 20.6 (33) |
| 1975 | *2.0 (7) | 2.6 (8) | 8.4 (24) | 12.3 (19) |
| 1976 | 0.3 (1) | 1.4 (4) | 5.7 (16) | 18.0 (27) |
| 1977 | 0.9 (3) | 2.3 (7) | 8.9 (25) | 15.1 (22) |
| 1978 | 1.4 (5) | *4.7 (15) | 6.7 (19) | 14.7 (21) |
| 1979 | 1.4 (5) | 3.5 (11) | *11.7 (33) | *22.0 (31) |
| 1980 | 1.4 (5) | 1.5 (5) | *10.7 (30) | 18.7 (26) |
| 1981 | *2.6 (9) | 3.3 (11) | *11.4 (32) | 16.3 (23) |

*Indicates the 5 years with the highest age- and sex-specific rates for the 24-year period. (4 highest years for 20–29 age group).

[1]Standardised for age by taking the average of the corresponding quinquennial rates for the age-ranges 20–24, 25–29, etc. to 50–54. (N.B. This happens to be equivalent, for these particular age-groups, to standardisation using the European or the World standard populations (Waterhouse *et al.*, 1976) in each age range).

[2]Defined using the following ICD codings:

7th revision (1958–67) 155.0
8th revision (1968–78) 155.0, 155.1 (155)
9th revision (1979–81) 155.0, 155.1

*Sources:* Registrar General. Statistical Reviews of England and Wales. London: HMSO 1958–1973. Office of Population Censuses & Surveys. Mortality Statistics-cause, England and Wales. Series DH2 nos. 1–7, London, HMSO 1974–1981.

deaths (Table IB), the fluctuations occurring over the whole of the time period considered (with sporadic clusters of high rates in the last 3 years for the 40–49 age group and in 1968–1970 for the 50–54 age group).

By contrast, among the female deaths (Table IA), there does appear to be a slight systematic increase in the mortality rates towards the end of the 1970s. This increase is most noticeable among the younger age groups. For each of the age groups 20–29, 30–39 and 40–49 the last 5 years have seen 3 of the 5 highest annual incidence rates in the entire 24-year period. It is difficult to interpret the results of any statistical test for trend in these data as the significance of the test will depend heavily on the year chosen as the starting point. If, however, one aggregates the two youngest and the two oldest age groups and compares the mortality rate for the last 6 years with that in the previous six (Table II), it appears that there has been a significant increase for younger women ($P<0.005$) but not for older women or for either age group in men.

**Table II** Comparison of age-standardised death rates from primary liver cancer between the years 1970–75 and 1976–81, for England and Wales.

| | *Death rates per $10^6$ (population at risk-millions)* 1970–75 | 1976–81 | $\chi^2$ (M.H.) | P |
|---|---|---|---|---|
| *Females* | | | | |
| 20–39 | 0.9 (38.02) | 1.8 (39.81) | 10.08 | <0.005 |
| 40–54 | 5.7 (27.08) | 5.9 (25.43) | 0.12 | N.S. |
| *Males* | | | | |
| 20–39 | 1.9 (38.99) | 2.0 (40.79) | 0.33 | N.S. |
| 40–54 | 10.7 (26.73) | 11.9 (25.50) | 1.68 | N.S. |

N.S. = not signifcant

The pill was introduced in England and Wales in the early 1960s and became common by the middle of that decade. In view of the long induction period that is commonly observed in human cancers attributable to chemicals, any carcinogenic effects attributable to the pill might be seen only after a substantial number of women had been exposed for 10–15 years i.e. towards the end of the 1970s. This is the pattern that has actually been observed. It might also be expected that any real effects would be more noticeable amongst women aged <40 years, for such women are likely to have been more heavily exposed to the pill, and, moreover, their very low rates of liver cancer before introduction of the pill should enable small absolute increases to be detected.

The data in Table I lend some support to the idea that oral contraceptives may have caused some cases of liver carcinoma, although the very low mortality figures among young women in 1981 (only 6 deaths for the 20–39 age group) suggest that the apparent trends among such women during the late 1970s may have been produced at least in part by the random fluctuation of small numbers.

If the increase in mortality is genuine, and due to a carcinogenic effect of the pill, similar trends should be apparent in other countries where the incidence of the disease is also normally low and pill consumption is equivalent to that in England and Wales. Four countries where this is the case are Australia, the United States, West Germany and the Netherlands which in 1975 had about 29, 16, 28 and 40% respectively, of women under 44 years on the pill compared with 20% in the UK (Population Reports, 1982). Corresponding triennial mortality rates for these 4 countries are given in Table III with the England and Wales figures for comparison. Although they do not extend until 1981, it is clear that in these countries there was no sign of the trends which were already evident by 1978 in young women in England and Wales. (The fluctuations seen in the data from Australia and the Netherlands probably reflect the smaller populations in these countries). Although there are certainly many possibilities for error in the allocation of deaths to liver tumours on death certificates it is remarkable that the rates recorded in the 5 countries in Table III are so similar. This similarity and our personal experience of the validity of the diagnosis, at least in youth and early middle age, suggests that in most developed countries such data will be reasonably trustworthy.

The evidence at present is therefore indecisive. If the risk of hepatocellular carcinoma associated with pill usage were as large as suggested by Henderson *et al.* (1983), then a clear increase in national mortality rates would be expected among young women. The only country of those considered where there is a suggestion of this, however, is England and Wales, which tends to suggest that any real effects must be either small or slow to appear. Moreover, even if the British figures were indicative of a real pill-related trend, the absolute risk of pill-induced liver carcinomas must still be small, probably involving no more than 10 deaths a year in the whole country among (in 1975) some 3.5 million users (Wiseman & Macrae, 1981). But, a risk that is small after only a few years of exposure may become substantial later; also, in countries where other risk factors (e.g. chronic active hepatitis B infection or aflatoxin contamination) are widespread the absolute risks may be much larger.

Apart from oral contraceptives (sales of which in Britain increased from 13 million packs in 1970 to 26 million packs in 1980), there are many other factors that might affect liver cancer trends: use of alcohol is increasing and parts of the growing homosexual community appear to be at unusual risk of hepatitis B transmission (as are some drug abusers). Also, among the plethora of chemicals that have come into widespread use during the past half century, there are many that can be oxidised more readily by the liver than by any other organ into highly reactive species (which is presumably why so many of the chemicals that have been found to cause cancer in animals affect the liver). In view of all this, it is somewhat reassuring that liver cancer remains such an uncommon cause of death in developed countries, and that clear increases are not generally apparent. These various factors, however, make it particularly important to continue to monitor trends in liver cancer mortality, as well as to monitor the causes of the disease directly.

We would like to thank Prof. M. Vessey for discussing this paper with us; Miss. K. Hughes for clerical assistance, and Miss. S. Jones for typing the manuscript.

**Table III** Triennial age-standardised[1] death certification rates per $10^6$ population for primary liver cancer[2] in England and Wales, USA, Australia, West Germany, and the Netherlands. 1967–69 to 1979–81.

| | *England & Wales* | *USA* | *Australia* | *W. Germany* | *The Netherlands* |
|---|---|---|---|---|---|
| *Females 20–39* | | | | | |
| 1967–69[3] | 1.1 | 1.4 | 0.6 | 1.0 | 0.7 |
| 1970–72 | 1.0 | 1.5 | 1.9 | 0.8 | 1.2 |
| 1973–75 | 0.9 | 1.3 | 0.7 | 0.9 | 1.3 |
| 1976–78 | 1.8 | 1.3 | 1.6 | 0.9 | 1.0 |
| 1979–81[4] | 1.8 | 1.5 | N/A | N/A | N/A |
| *Females 40–54* | | | | | |
| 1967–69[3] | 5.5 | 6.3 | 6.2 | 7.5 | 5.7 |
| 1970–72 | 5.2 | 6.2 | 6.6 | 8.9 | 5.2 |
| 1973–75 | 6.1 | 5.9 | 4.5 | 8.1 | 10.3 |
| 1976–78 | 4.5 | 6.7 | 6.5 | 7.3 | 4.7 |
| 1979–81[4] | 7.4 | 6.6 | N/A | N/A | N/A |
| *Males 20–39* | | | | | |
| 1967–69[3] | 1.8 | 1.4 | 1.0 | 1.2 | 1.4 |
| 1970–72 | 1.9 | 2.1 | 2.7 | 1.5 | 1.1 |
| 1973–75 | 1.8 | 1.8 | 1.1 | 1.1 | 1.9 |
| 1976–78 | 1.8 | 1.9 | 2.1 | 1.3 | 2.9 |
| 1979–81[4] | 2.3 | 2.0 | N/A | N/A | N/A |
| *Males 40–54* | | | | | |
| 1967–69[3] | 12.9 | 13.8 | 12.6 | 12.2 | 17.2 |
| 1970–72 | 10.6 | 12.3 | 14.4 | 13.8 | 11.8 |
| 1973–75 | 10.9 | 11.6 | 9.1 | 14.9 | 14.1 |
| 1976–78 | 10.0 | 13.7 | 14.0 | 14.1 | 13.6 |
| 1979–81[4] | 13.9 | 12.0 | N/A | N/A | N/A |

N/A = not available

[1]Standardisation as for England and Wales rates in **Table I**, then 3 years averages calculated.

[2]Defined as in **Table I.**

[3]Figures for USA, Australia, and West Germany for 1968 and 1969 only.
Figures for the Netherlands for 1969 only.

[4]Figures for USA for 1979 only. The lack of data for Australia, West Germany, and the Netherlands in this period is due to the introduction of the 9th revision ICD coding which combines the category "liver cancer, unspecified primary or secondary" within the 155 rubric. This means that for countries which only publish data using 3-digit ICD classifications, it is impossible to compare rates from primary liver cancer beyond the periods when the 8th revision ceased being used.

*Sources:* England and Wales—as in **Table I.**

USA—Deaths "Vital statistics of the United States: Volume II; Mortality (Part A) US Govt. Printing Office, Washington D.C. 1968–1978, Deaths for 1979 and Populations—Personal communication from US Bureaus of the census. Populations correlated for census undercount as in Doll & Peto (1981), Appendix B.

Australia—"Australia—Causes of Death" Commonwealth Bureaus of Census and Statistics, Canberra 1968–73. "Causes of Death—Australia" Australian Bureaus of Statistics, Canberra 1974–1978.

West Germany—Deaths "Gesundheitswesen—Reihe 4—Todesurachen" Populations—"Statistics Jahrbuch für die Bundesrepublik Deutschland. 1968–1978 Statistics Bundesamt, Wiesbaden.

Netherlands—Deaths "Netherlands—causes of death", Populations "Statistical Yearbook of the Netherlands" 1969–1978 Netherlands Central Bureau of Statistics.

### References

ALDERCREUTZ, H. & TENHUNEN, R. (1970). Some aspects of the interaction between natural and synthetic female sex hormones and the liver. *Am. J. Med.*, **49**, 630.

BAUM, J.K., HOLTZ, F., BOOKSTEIN, J.J., & KLEIN, E.W. (1973). Possible association beteen benign hepatomas and oral contraceptives. *Lancet*, **ii**, 926.

CHRISTOPHERSON, W.M., MEYS, E.T., & BARROWS, G. (1978). Hepatocellular carcinoma in young women on oral contraceptives. *Lancet*, **2**, 38.

COMMITTEE ON SAFETY OF MEDICINES (1972). *Carcinogenicity Tests of Oral Contraceptives*. London: H.M.S.O.

DAVIS, M., PORTMAN, B., SEARLE, M., WRIGHT, R. & WILLIAMS, R. (1975). Histological evidence of carcinoma in a hepatic tumour associated with oral contraceptives. *Br. Med. J.*, **4**, 496.

DOLL & PETO (1981). *The Causes of Cancer*. Oxford: O.U.P. p. 1268.

EDMONDSON, H.A., HENDERSON, B., & BENTON, B. (1976). Liver cell adenomas associated with the use of oral contraceptives. *N. Engl. J. Med.*, **294**, 470.

GALA, K.V., & GRIFFIN, T.W., (1983). Hepatomas in young women on oral contraceptives: Report of two cases and review of the literature. *J. Surg. Oncol.*, **22**, 11.

HENDERSON, B.E., PRESTON-MARTIN, S., EDMONDSON, H.A., PETERS, R.L., & PIKE, M.C. (1983). Hepatocellular carcinoma associated with use of oral contraceptives. *Br. J. Cancer*, **48** (this issue).

KLATSKIN, G. (1977). Hepatic tumours: possible relation to use of oral contraceptives. *Gastroenterology*, **73**, 386.

METTLIN, L. & NATARAJAN, N. (1981). Studies on the role of oral contraceptive use in the etiology of benign and malignant liver tumours. *J. Surg. Oncol.*, **18**, 73–85.

NEUBERGER, J., PORTMAN, B., NUNNERLEY, H.R., LAWS, J.W., DAVID, M. & WILLIAMS, R. (1980). Oral contraceptive-associated liver tumours: Occurence of malignancy and difficulties in diagnosis. *Lancet*, **i**, 273.

POPULATION REPORTS, ORAL CONTRACEPTIVES. (1982). *Series A*, **6**, Baltimore: Population Information Program.

ROOKS, J.B., ORY, H.W., ISHAK, K.G. & 4 others (1979). Epidemiology of hepatocellular adenoma *J.A.M.A.*, **242**, 644.

SHAR, S.R. & KEW, M.C. (1982). Oral contraceptives and hepatocellular carcinoma. *Cancer*, **49**, 407.

VANA, J., MURPHY, G.P., ARONOFF, B.L. & BAKER, H.W. (1977). Primary liver tumour and oral contraceptives. Results of a survey. *J.A.M.A.*, **238**, 2154.

WANLESS, I.R. & MEDLINE, A. (1982). Role of estrogens as promoters of hepatic neoplasia. *Lab. Invest.*, **46**, 313.

WATERHOUSE, J., MUIR, C., CORREA, P. & POWELL, J. (eds) (1976). *Cancer Incidence in Five Continents*, **Vol. III** Lyon: International Agency for Research on Cancer.

WISEMAN, R.A. & MACRAE, K.D. (1981). Oral contraceptives and the decline in mortality from circulatory disease. *Fertil. Steril.*, **35**, 277.

WORLD HEALTH ORGANISATION TECHNICAL REPORT (1978). Series 619. *Steroid Contraception and the Risk of Neoplasia*. Geneva: W.H.O.

YARGER, J.D. & YARGER, R. (1980). Oral contraceptive steroids as promoters of hepatocarcinogenesis in female Sprague-Dawley rats. *Cancer Res.*, **40**, 3680.